# Peroxiredoxin 5 Protects TGF-β Induced Fibrosis by Inhibiting Stat3 Activation in Rat Kidney Interstitial Fibroblast Cells

**DOI:** 10.1371/journal.pone.0149266

**Published:** 2016-02-12

**Authors:** Hoon-In Choi, Seong Kwon Ma, Eun Hui Bae, JongUn Lee, Soo Wan Kim

**Affiliations:** 1 Department of Internal Medicine, Chonnam National University Medical School, Gwangju, Republic of Korea; 2 Department of Physiology, Chonnam National University Medical School, Gwangju, Republic of Korea; University of Birmingham, UNITED KINGDOM

## Abstract

Renal fibrosis is a common final pathway of end-stage kidney disease which is induced by aberrant accumulation of myofibroblasts. This process is triggered by reactive oxygen species (ROS) and proinflammatory cytokines generated by various source of injured kidney cells. Peroxiredoxin 5 (Prdx5) is a thiol-dependent peroxidase that reduces oxidative stress by catalyzing intramolecular disulfide bonds. Along with its antioxidant effects, expression level of Prdx5 also was involved in inflammatory regulation by immune stimuli. However, the physiological effects and the underlying mechanisms of Prdx5 in renal fibrosis have not been fully characterized. Sprague-Dawley rats were subjected to unilateral ureteral obstruction (UUO) for 1 or 7 days. For the *in vitro* model, NRK49F cells, a rat kidney interstitial fibroblast cell lines, were treated with transforming growth factor β (TGF-β) for 0, 1, 3, or 5 days. To access the involvement of its peroxidase activity in TGF-β induced renal fibrosis, wild type Prdx5 (WT) and double mutant Prdx5 (DM), converted two active site cysteines at Cys 48 and Cys 152 residue to serine, were transiently expressed in NRK49F cells. The protein expression of Prdx5 was reduced in UUO kidneys. Upregulation of fibrotic markers, such as fibronectin and alpha-smooth muscle actin (α-SMA), declined at 5 days in time point of higher Prdx5 expression in TGF-β treated NRK49F cells. The overexpression of wild type Prdx5 by transient transfection in NRK49F cells attenuated the TGF-β induced upregulation of fibronectin and α-SMA. On the other hand, the transient transfection of double mutant Prdx5 did not prevent the activation of fibrotic markers. Overexpression of Prdx5 also suppressed the TGF-β induced upregulation of Stat3 phosphorylation, while phosphorylation of Smad 2/3 was unchanged. In conclusion, Prdx5 protects TGF-β induced fibrosis in NRK49F cells by modulating Stat3 activation in a peroxidase activity dependent manner.

## Introduction

Aberrant activation of fibroblasts to myofibroblasts is one of the hallmarks of renal fibrosis in chronic kidney diseases such as diabetes mellitus and hypertension. Activated myofibroblasts lead to accumulation of extracellular matrix, such as alpha-smooth muscle actin (α-SMA), fibronectin, and vimentin. This process is triggered by reactive oxygen species and inflammatory cytokines generated in injured kidney resident cells [1–4]. Transforming growth factor β (TGF-β) is recognized as a major pro-fibrotic cytokine of renal fibrosis. During renal fibrosis, TGF-β1 exerts its biological and pathological activities via Smad-dependent and Smad-independent signaling pathways. In canonical TGF-β/Smads pathway, the binding of TGF-β1 to its receptor II (TβRII) activates the TGF-β receptor type I (TβRI) kinase. Then TβRI phosphorylates Smad2 and Smad3 and subsequently, phosphorylated Smad2/Smad3 bind to Smad4 to form the Smad complex. This complex then translocates into the nucleus to regulate the fibrotic marker gene transcription, including type I collagen, α-SMA [5–7]. In non-canonical TGF-β/Smad pathway, TGF-β utilizes a multiple signaling pathway to regulate fibrotic gene expression through MAPKs pathway, Rho-like GTPase signaling pathways, phosphatidylinositol-3-kinase/AKT-mTOR pathway, and Jak-Stat pathway [8–10].

Peroxiredoxin 5 (Prdx5) is an atypical member of the peroxiredoxin family that reduces hydrogen peroxide, peroxynitrite, and alkylhydroperoxide by catalyzing intramolecular disulfide formation in a conserved peroxidatic N-terminal cysteine (Cys48) and a resolving C-terminal cysteine residue (Cys152). It is broadly localized in the cytosol, nucleus, mitochondria, and peroxisome, and performs specific functions according to its subcellular localization [11]. Expression of Prdx5 is mainly regulated by inflammatory stimuli or inflammatory diseases rather than by direct oxidants such as hydrogen peroxide and paraquat. Prdx5 is up-regulated in lipopolysaccharide-stimulated macrophages or microglial cells to provide anti-oxidative and anti-inflammatory protection against oxidative stress [12–15]. Up-regulation of Prdx5 has also been reported in osteoarthritic cartilage and in TNF-α or IL-1β treated cartilage explants from patients with osteoarthritis [16]. This upregulation disrupts Wnt/β-catennin pathway regulation, leading to cartilage loss [17]. Despite the association of Prdx5 with inflammatory regulation, the physiological effects of Prdx5 in renal fibrosis have not been fully characterized, and the underlying mechanisms remain poorly understood.

Unilateral ureteral obstruction (UUO) is a well-established renal injury model that reflects inflammatory and fibrotic pathophysiology of chronic obstructive nephropathy [18]. In this study, we demonstrated the association between Prdx5 expression and renal fibrosis. Prdx5 is dramatically reduced in UUO versus control kidneys, but is gradually increased in TGF-β treated NRK49F cells, a fibroblast-like proximal tubule cells, according to TGF-β induced ROS generation. To determine whether Prdx5 functions as a pro-fibrotic or anti-fibrotic factor, Prdx5 was transiently expressed in NRK49F cells. Ectopic expression of Prdx5 attenuated expression of the pro-fibrotic markers, such as fibronectin and α-SMA, in a peroxidase activity-dependent manner. Prdx5 also preferentially delayed activation of Stat3, a transcriptional activator of fibrotic gene expression, but had not effect on Smad2/3 activation. These results suggest Prdx5 is one of anti-fibrotic effectors in renal fibrosis.

## Materials and Methods

### Animals

The animal experiments were approved by the Animal Care Regulations (ACR) Committee of Chonnam National University Medical School and our protocols conformed to the institution guidelines for experimental animal care and use. Experiments were performed using male Sprague-Dawley rats (180~200 g, Samtako, Korea). Rats were housed under controlled temperature (21±2°C) in a 12 h light-dark cycle. Unilateral ureteral obstruction was induced by ligation of the left ureter for one day (n = 4) or for seven days (n = 4). The abdominal cavity was opened, and 2–0 silk ligature was placed at left proximal ureter under anesthesia with ketamine (50 mg/kg, intraperitoneally; Yuhan, Seoul, Korea). The control group for one day (n = 4) and seven days (n = 4) received the same treatment, with the exception of the ligature. The rats had free access to standard rat feed and tap water and were sacrificed by decapitation on day 1 or day 7 after the operation. The kidney was rapidly removed, dissected into the cortex/outer stripe of the outer medulla, inner stripe of outer medulla and inner medulla, and processed for western blotting.

### Cell culture and TGF-β treatment

NRK49F cells, a rat kidney interstitial fibroblast cell line (ATCC, Manassas, VA), were cultured in complete DMEM/low glucose media (WelGene, Daegu, Korea) supplemented with 10% FBS, 50 U/mL penicillin and 50 μg/mL streptomycin at 37°C under a humidified 5% CO_2_ atmosphere. For TGF-β treatment, cells were starved for one day with DMEM/low glucose media containing 0.5% FBS and were treated with 10 ng/ml TGF-β for an indicated time with same media in all experiments.

### Reagents and Antibodies

Recombinant human TGF-β1 was purchased from R&D Systems (Minneapolis, MN). Fugene HD transfection reagent was from Promega (Madison, WI). Antibodies against phospho-Stat3 (Tyr705), Total-Stat3, Total-Smad2/Smad3, Phospho-Smad2 (Ser465/467)/Smad3 (Ser423/425), TGF-β, Total-Jak2, and Phospho-Jak2 were all from Cell Signaling Technology (Danvers, MA). Fibronectin was from Santa Cruz (Dallas, Texas). E-cadherin was purchased from BD Biosciences (Franklin Lakes, NJ). Antibodies against α-SMA and β-actin were from Sigma-Aldrich (St. Louis, MO). Specific antibodies against Prdx1, Prdx2, Prdx3, Prdx4, Prdx5, and Prdx6 were gifts from Dr. Ho Zoon Chae (Chonnam National University, Korea).

### Plasmid construction

The *full-length cDNA* of rat Prdx5 containing the mitochondrial target sequence was obtained from NRK49F *cDNA* by PCR amplification with a forward primer containing a *Xho*I site (*5'-GTTCTCGAGATGGTCCAGCTGAGG-3'*) and a reverse primer containing a *Mlu*I site (*5'-CAAACGCGTTCAGAGTTGTGAGAG-3'*). To produce wild-type Prdx5 protein in mammalian cells, the PCR product was digested with *Xho*I and *Mlu*I, then ligated into *pCR3*.*1-MAR* [19]. To produce *double mutant Prdx5 (DM)*, *Cys 48* and *Cys 152* were replaced with *Ser* by site-directed PCR-mediated mutagenesis. In first, the *C48S* substitution was generated with forward primer (*5'-TTACACCTGGCTCATCCAAGACCC-3'*) and reverse primer (*5'-TGGGTCTTGGATGAGCCAGGTGTAA-3'*) and the substituted PCR products were subcloned into *pCR3*.*1-MAR* vector digested with same restriction enzyme, like as *WT Prdx5*. Next, the *C152S* mutation was serially substituted in the *C48S Prdx5* as a template with forward primer (*5'-GTTCTCGAGATGGTCCAGCTGAGG-3'*) and reverse primer (*5'-CAAACGCGTTCAGAGTTGTGAGAGGATGTTGGGGGCCAGGCTGCTGGTGA-3'*). Double mutants PCR products were finally subcloned to same vector. The plasmid constructs were confirmed by sequencing.

### Transient expression

NRK49F cells were seeded into 60 mm dishes at 2 x 10^5^ per plate. After 1 day, the cells were transfected with 3 μg of plasmid DNA using Fugene HD transfection reagent was from Promega (Madison, WI) by 1:3 ratio of DNA to transfection reagent. After 1 day transfection, the cells were starved with earlier mentioned medium for another one day, followed by TGF-β treatment. The expression levels were checked by western blotting.

### Cell fractionation

To check the subcellular localization of Prdx5 induced by TGF-β in NRK49F cells, 2 x 10^5^ of NRK49F cells were treated with 10 ng/ml of TGF-β. The cells were separated to cytosol, mitochondria, and nucleus with cell fractionation kit (Abcam, ab109719, Cambridge,UK), based on sequential detergent-extraction method without the need for mechanical disruption. The separated cytosol, mitochondria, and nucleus samples were evaluated with GAPDH, Prdxs3, and Histone H3, respectively.

### Measurement of intracellular ROS

Levels of intracellular reactive oxygen species (ROS) were assessed using 5,6-chlorommethyl-2’,7’-dichlorodihydrofluorescein diacetate (Invitrogen). NRK49F cells were seed into 12 well or 48 well plates at 5 x 10^4^ or 2.5 x 10^4^ per each well. The cells were changed with phenol red-free medium containing with 0.5% FBS and treated with 10 ng/ml of TGF-β for an indicated time at 37°C under a humidified 5% CO_2_ atmosphere, after which they were washed with Hank’s buffered salt solution (HBSS). The cells were washed once with HBSS, and incubated with 10 μM CM-H_2_DCFDA (Invitrogen, Carlsbad, CA, USA) for 20 min at 37°C. Thereafter, the cells were washed with phenol red-free DMEM/low glucose medium containing 0.5% FBS, and added with same medium. Finally, the cells were analyzed using fluorescence microscope (Nikon ECLIPSE TE2000) and fluorescence microplate reader (Gemini XPS Microplate Reader) to quantify the fluorescent of oxidized DCFH.

### Western blotting

Kidney tissue was homogenized in ice-cold lysis buffer [0.3 M sucrose, 25 mM imidazole, 1 mM EDTA, 8.5 μM leupeptin, and 1 mM PMSF (pH 7.2)]. Tissue homogenates were centrifuged at 1500 *g* for 20 min at 4°C to remove cell debris. NRK49F cells were lysed with RIPA buffer containing 50 mM Tris-HCl pH 7.5, 0.15 M NaCl, 1% Triton X-100, 0.5% sodium deoxycholate, 0.1% SDS, 1 mM NaF, 1 mM Na_3_VO_4_, 0.1 mM AEBSF, 0.5 μg/mL leupeptin, and 1 mM PMSF. Cell homogenates were centrifuged at 15000 *g* for 30 min at 4°C to remove cell debris. Total protein concentration was determined by bicinchoninc acid (BCA) assay (Pierce, Rockford, IL). Proteins were separated in 8 or 13.5% polyacrylamide gels, transferred to a PVDF membrane, and incubated with the indicated primary antibodies. The membranes were then incubated with secondary anti-rabbit or anti-mouse horseradish peroxidase-conjugated antibodies and visualized with an enhanced chemiluminescence system (Santa Cruz, Dallas, Texas).

### Statistical analysis

Data are expressed as means ± SEM. An unpaired t-test was used to identify differences between groups. Multiple comparisons were made by one-way ANOVA and post hoc Tukey HSD test. Differences with values of *p* < 0.05 were considered significant.

## Results

### Prdx5 is associated with renal fibrosis

**Fig 1:**
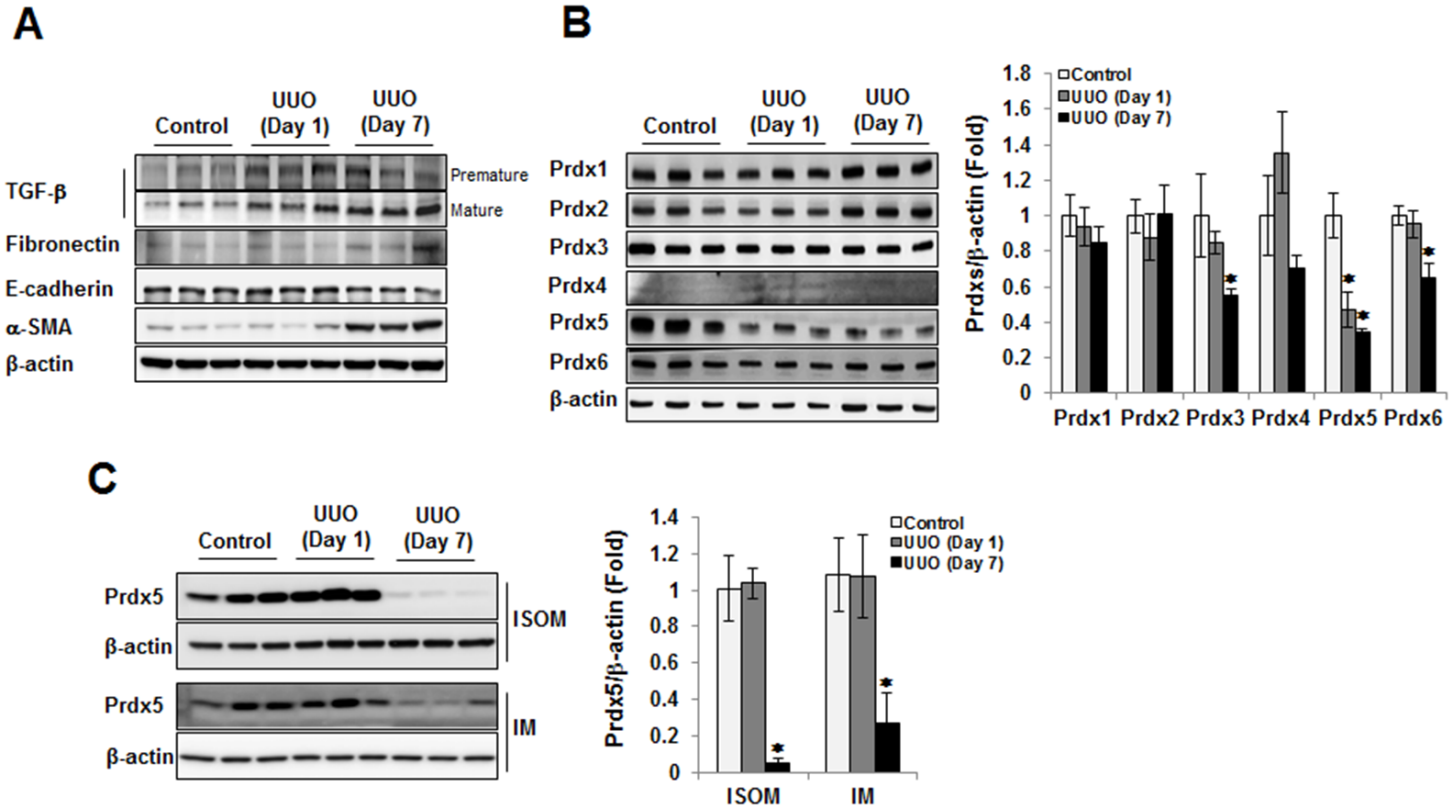
The protein levels of Prdxs in UUO kidney. To check of progression level of renal fibrosis in left kidney during ureter obstruction, we analyzed the expression of fibrotic marker proteins (premature and mature form of TGF-β, fibronectin, and α-SMA) and epithelial marker protein (E-cadherin) (A). To assess the involvement of Prdxs in renal fibrosis, we analyzed the expression of several Prdx isotypes (Prdx1, Prdx2, Prdx3, Prdx4, Prdx5, and Prdx6) in the cortex/outer stripe of the outer medulla tissue homogenate UUO kidney. Bar graphs show mean Prdxs/β-actin expression as measured by densitometry (B). To further understand the involvement of Prdx5 in renal fibrosis, we analyzed the expression level of Prdx5 in the inner stripe of the outer medulla (ISOM) and inner medulla (IM). Bar graphs show mean Prdx5/β-actin expression as measured by densitometry (C). *p < 0.05 Day 1 and Day 7 vs. control kidney

We developed a rat UUO model of renal fibrosis by ligation of the left ureter for one or seven days. Progressive renal fibrosis was monitored by measuring levels of pro-fibrotic mediator such as premature or mature form of TGF-β, and the increase of myofibroblast marker proteins such as fibronectin and α-SMA, and the decrease of the epithelial marker such as E-cadherin. Premature form of TGF-β expression was gradually increased at 1 day and mature form of TGF-β expression was highly sustained at 7 days after ureter obstruction. The expression of fibronectin and α-SMA were increased at 7 days, and also E-cadherin was reversely decreased at 7 days (Fig 1A). To determine involvement of Prdxs in renal fibrosis, we analyzed the expression pattern of Prdxs isotypes (Prdx1, Prdx2, Prdx3, Prdx4, Prdx5, and Prdx6) in the cortex/outer stripe of outer medulla of UUO kidney. Among the isotypes, mitochondrial forms of Prdxs (Prdx3 and Prdx5) and Prdx6 were decreased with the progression of renal fibrosis. On the other hand, the protein expression of Prdx1, Prdx2 and Prdx4 was not significantly changed in UUO kidney. Expression of Prdx5 was rapidly down-regulated in one day UUO kidney, the early phase of fibrosis (Fig 1B). Likewise reduction of Prdx5 expression in the cortex/outer stripe of the outer medulla of UUO kidney, the protein level of Prdx5 were also down-regulated in the inner stripe of outer medullar and inner medulla in accordance with fibrosis progression (Fig 1C). These results suggest the involvement of Prdx5 in renal fibrosis.

**Fig 2:**
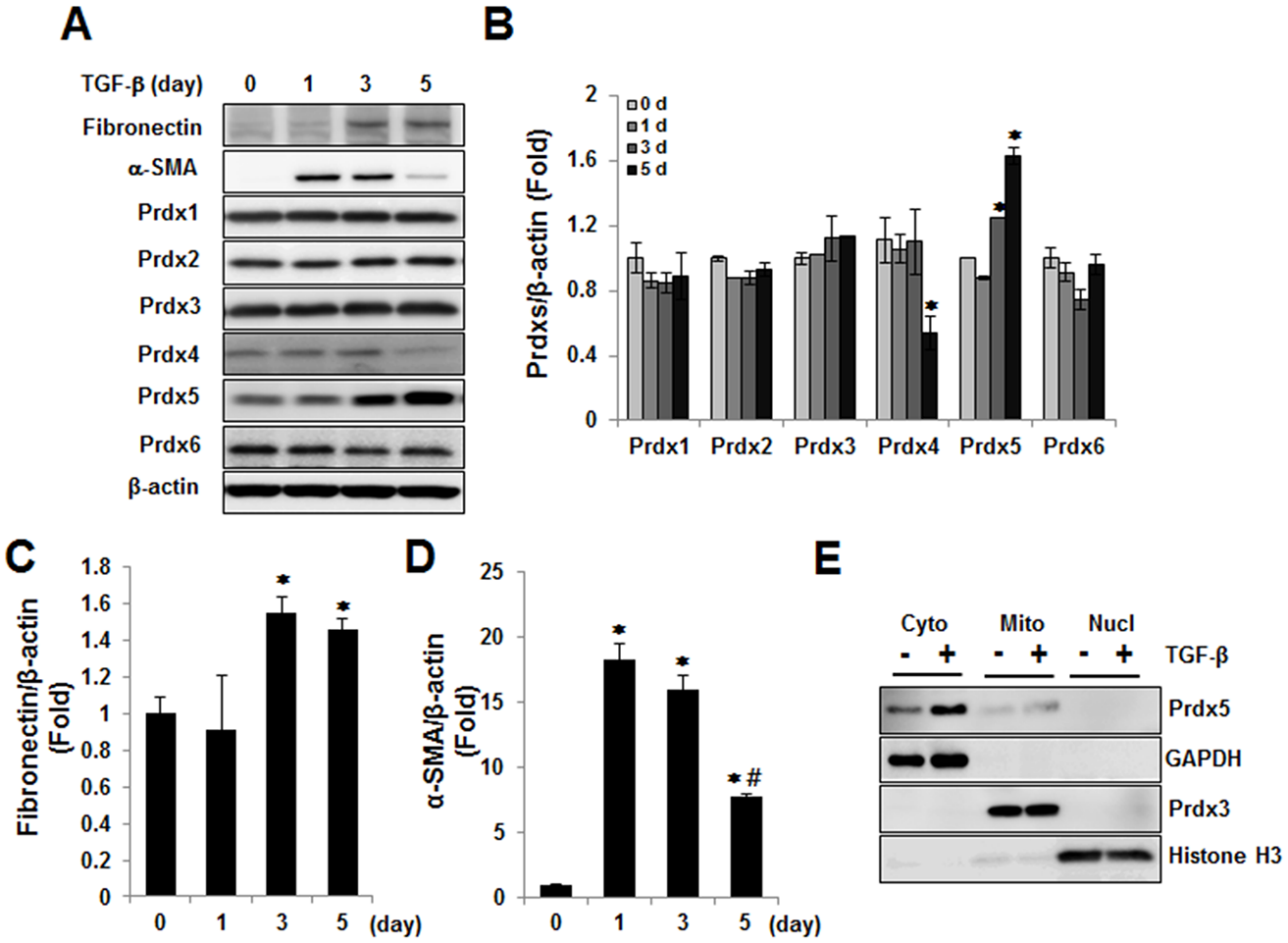
Up-regulation of cytosolic Prdx5 in TGF-β treated NRK49F cells. NRK49F cells were exposed to TGF-β. Expression of Prdx proteins (Prdx1, Prdx2, Prdx3, Prdx4, Prdx5, and Prdx6) and fibrotic markers (fibronectin and α-SMA) was analyzed by western blotting (A). Bar graphs show mean Prdxs, fibronectin, and α-SMA/β-actin expression as measured by densitometry (B-D). Subcellular localization of Prdx5 elicited by TGF-β was analyzed with fractionation sample (cytosol, mitochondria, and nucleus), mentioned as Materials and Methods. GAPDH, Prdx3, and Histone H3 were used to fractionation control markers for cytosol (Cyto), mitochondria (Mito), and nucleus (Nuvl), respectively (E). *p<0.05 Day 1 and Day 7 vs. control kidney; *p <0.05 Day 0 vs. Day 3; ^#^*p* < 0.05 Day 3 vs. Day 5

To verify the involvement of Prdx5 in renal fibrosis, NRK49F cells were treated with TGF-β for 0, 1, 3, and 5 days. The protein level of α-SMA peaked at 1 day, and the protein level of fibronectin peaked at 3 days and then subsequently decreased at 5 days. Interestingly, protein expression of Prdx5 dramatically changed over the course of TGF-β treatment among Prdxs isotypes. The level of Prdx5 gradually increased until 5 days after TGF-β treatment, while α-SMA and fibronection declined at 5 days after the peak 3 days (Fig 2A–2D). In association with these changes, other Prdxs isotypes remained unchanged except that Prdx4 expression was decreased at 5 day of TGF-β treatment (Fig 2A and 2B).

To determine the subcellular localization of Prdx5 increased by TGF-β treatment in NRK49F cells, we next separated the lysates into cytosol, mitochondria, and nucleus fractions. We found that Prdx5 was significantly increased in the cytosol fraction (Fig 2E). These results suggest that the cytosolic Prdx5 is associated with pathophysiology of renal fibrosis although the difference between down-regulation of Prdx5 in UUO-induced renal fibrosis and up-regulation of Prdx5 in TGF-β treated NRK49F cells was further elucidated.

### Prdx5 expression is dependent on TGF-β induced ROS generation

**Fig 3:**
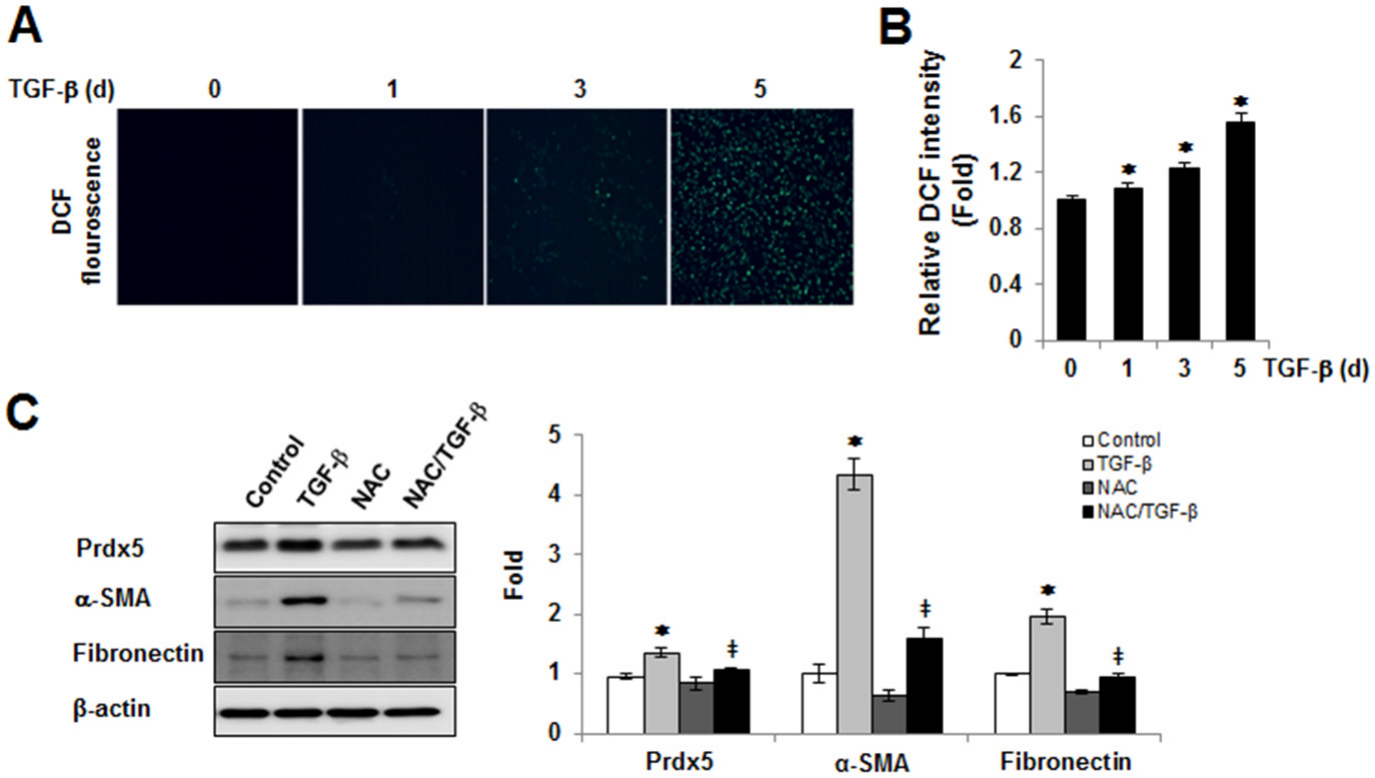
The role of the intracellular ROS in TGF-β induced Prdx5 up-regulation. To assess TGF-β mediated intracellular ROS levels, ROS probe (10 μM CM-H_2_DCFDA, Invitrogen) were loaded in TGF-β treated NRK49F cells for 0, 1, 3, and 5 days. Intracellular ROS levels were analyzed using fluorescence microscope (Nikon ECLIPSE TE2000) (A). Bar graphs show relative ROS levels as measured by fluorescence microplate reader (Gemini XPS Microplate Reader) (B). To assess the role of ROS in TGF-β induced Prdx5 up-regulation, NRK49F cells were incubated for 3 day with TGF-β in the presence or absence of 10 mM NAC. The protein levels of Prdx5, fibronectin, and α-SMA were assayed using western blotting (C). *p<0.05 TGF- β treated 1, 3, and 5 day vs. control 0 day

Reactive oxygen species (ROS) are known to act as one of mediators in progression of renal fibrosis [20]. Multiple injury stimuli, such as transforming growth factor β1 (TGF-β1), tumor necrosis factor alpha (TNF-α), platelet derived growth factor (PDGF), angiotensin II (ANG II), hyperglycemia, oxidized low-density lipoprotein (oxLDL), albumin, and lipopolysaccharide (LPS) were affected to increase intracellular ROS production through by the increase of activity or expression of NADPH oxidase [21–24]. To test whether ROS signaling was associated with TGF-β induced Prdx5 up-regulation in NRK49F cells, we tested the effect of NAC, a chemical antioxidant, in TGF-β induced Prdx5 up-regulation. Consistent with an earlier report [25], DCF florescence was increased at TGF-β treated NRK49F cells in time-dependent manner (Fig 3A and 3B). Up-regulation of Prdx5 by TGF-β was reduced by NAC treatment, resulting in antioxidant effect of NAC lead to reduce expression of the fibrotic markers (Fig 3C). These results suggest that Prdx5 is induced by TGF-β mediated ROS generation.

### Prdx5 protects TGF-β induced fibrosis

**Fig 4:**
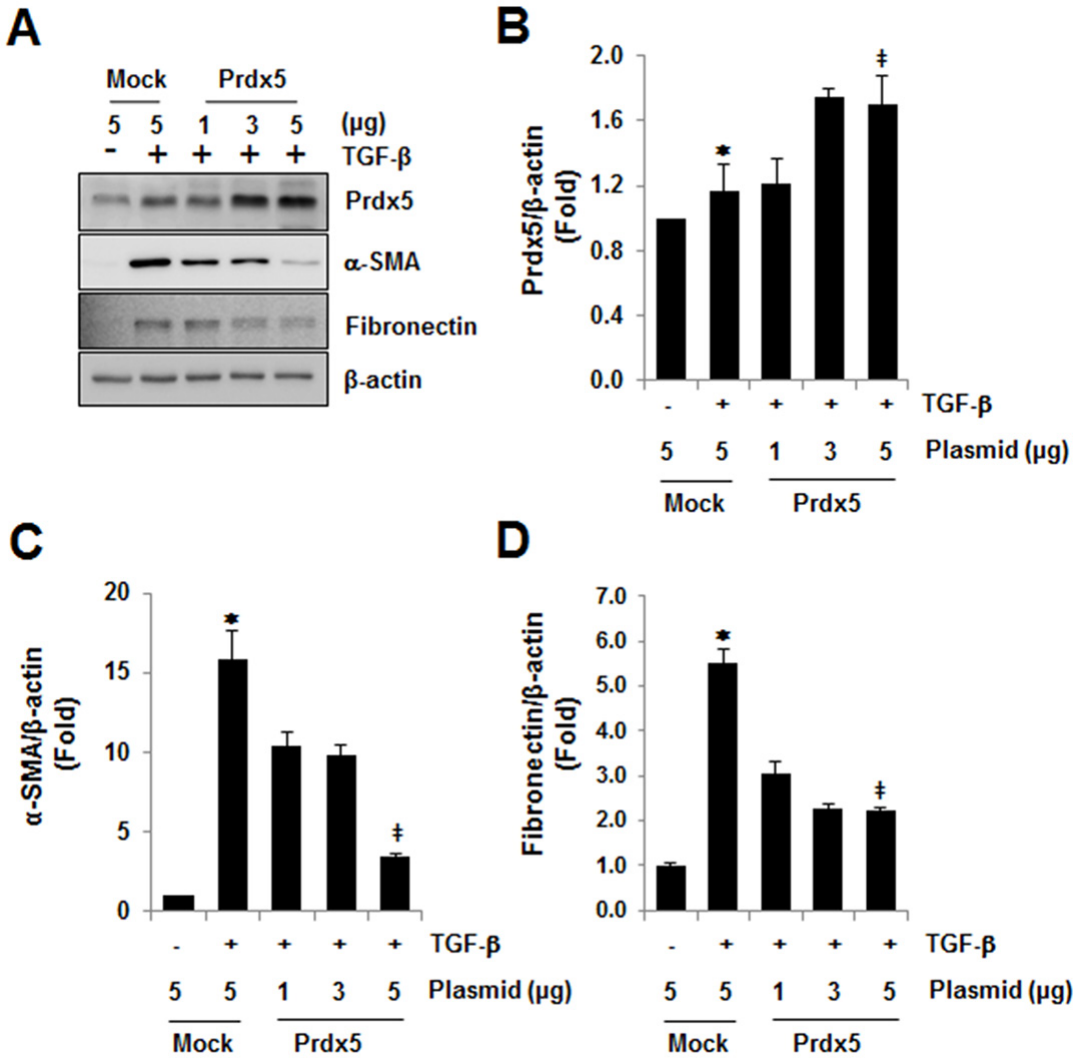
Anti-fibrotic effect of Prdx5 in TGF-β induced renal fibrosis. NRK49F cells were transiently transfected with various amounts of *Wild-type Prdx5*. After one day, cells were starved with DMEM/low glucose media with 0.5% FBS for one day, and then exposed to TGF-β for three days. Changes in expression of fibrotic marker proteins by Prdx5 over-expression were measured (A). Bar graphs show mean Prdx5/β-actin (B), α-SMA/β-actin (C), and fibronectin/β-actin expression (D) as measured by densitometry. *p < 0.05, TGF-β treated *Mock* vs. untreated *Mock*; ^‡^ *p* < 0.05, TGF-β treated *Mock* vs. *WT Prdx5*

The next aim was to determine whether Prdx5 functions as a pro-fibrotic or anti-fibrotic factor in the progression of fibrosis. A plasmid encoding *Prdx5* was transiently transduced into NRK49F cells, and then treated with TGF-β for 3 days in the peak time of fibrotic marker gene expression. Ectopic Prdx5 was expressed in dose-dependent manner (Fig 4A and 4B). Treatment of TGF-β was induced to expression of fibrotic markers α-SMA and fibronectin, but this effect was reversed by overexpression level of Prdx5 (Fig 4A, 4C and 4D). These results indicate that Prdx5 function as anti-fibrotic effector to protect fibrosis.

### Prdx5 regulates TGF-β induced fibrosis in its peroxidase activity dependent manner

**Fig 5:**
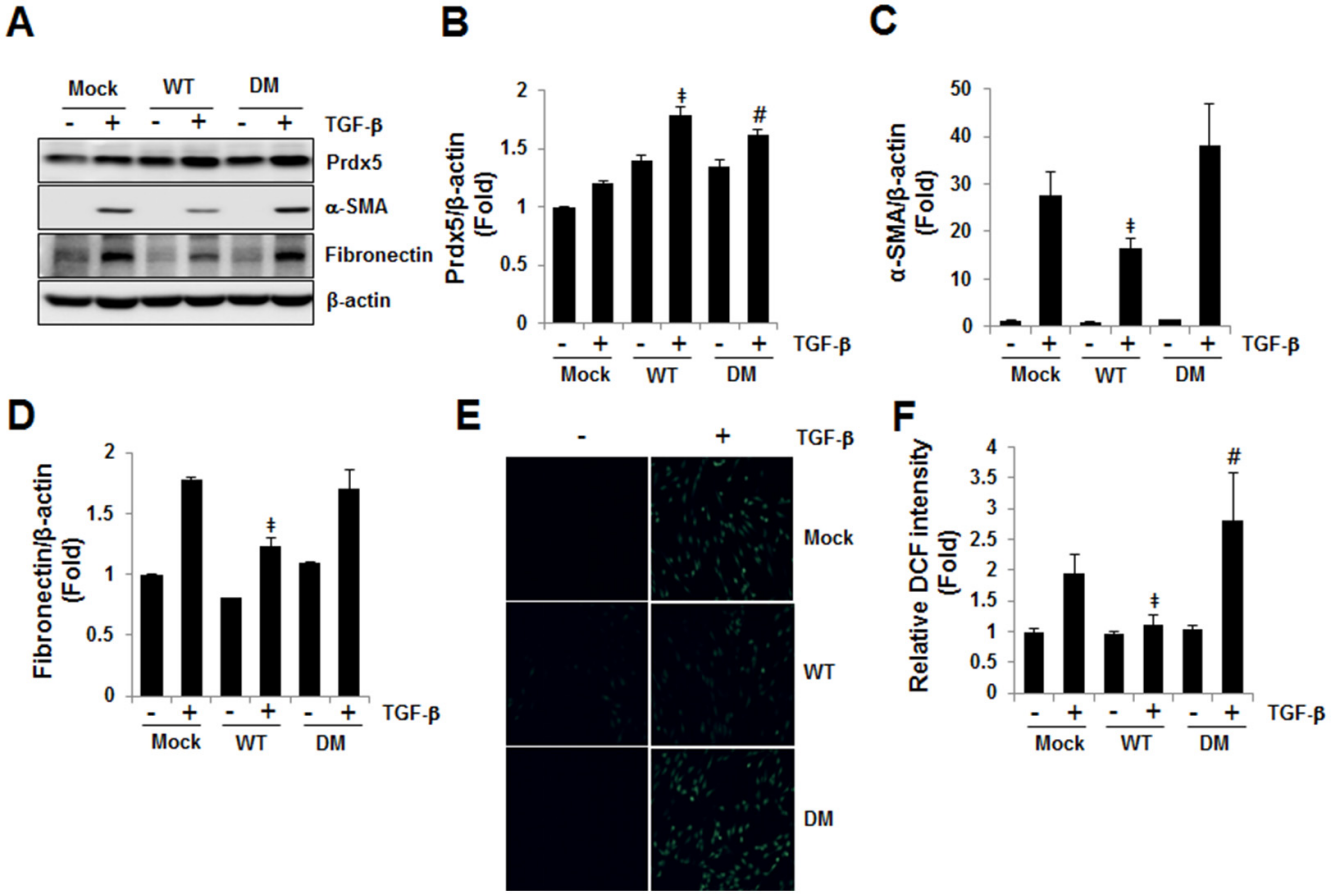
Prdx5 regulatory efficacy is peroxidase activity-dependent. The anti-fibrotic activities of *wild-type* (*WT*) and *double mutant Prdx5* (*DM*; *Cys48Ser* and *Cys152Ser*) were evaluated after transient transfection of NRK49F cells, which were starved for one day, then exposed to TGF-β for three days. Protein expression of fibrotic markers proteins was compared between cells expressing *WT* and *DM Prdx5* vs. *Mock*-transfected cells (A). Bar graphs show mean Prdx5/β-actin (B), α-SMA/β-actin (C), and fibronectin/β-actin expression (D) as measured by densitometry. To assay ROS levels by WT Prdx5 or DM Prdx5 expression, the intracellular ROS levels were detected with fluorescence microscope (Nikon ECLIPSE TE2000) (E) and the relative fluorescent of oxidized DCFH was detected by fluorescence microplate reader (Gemini XPS Microplate Reader) (F). ^‡^*p* < 0.05; TGF-β treated *Mock* vs. *WT*; ^#^*p* < 0.05; TGF-β treated *Mock* vs. *DM*

Prdx5 is an atypical 2-Cys Prdxs, whose activity is catalyzed by intramolecular disulfide formation at the conserved Cys48 and Cys152 residues [11]. To determine whether the peroxidase activity of Prdx5 is required for its anti-fibrotic activity in TGF-β induced fibrosis, we constructed a *double mutant* (*DM Prdx5*) in which the catalytic *Cys48* and *Cys152* of *Prdx5* were substituted with *Ser*. In consistent to Fig 4, *wild type Prdx5 (WT)* attenuated up-regulation of fibronectin and α-SMA compared to *Mock* at 3 days of TGF-β treatment, while *double mutant Prdx5 (DM)* did not affect the TGF-β induced expression of fibronectin and α-SMA compared to *Mock* (Fig 5A–5D). In fact, *WT Prdx5* effectively reduced ROS level elicited by TGF-β, while *DM Prdx5* increased ROS level compared to *Mock*-transfected cells (Fig 5E and 5F). Taken together, these results imply that Prdx5 protects TGF-β induced fibrosis in its peroxidase activity dependent manner associated with antioxidant effects.

### Prdx5 negatively modulates TGF-β induced Stat3 activation

**Fig 6:**
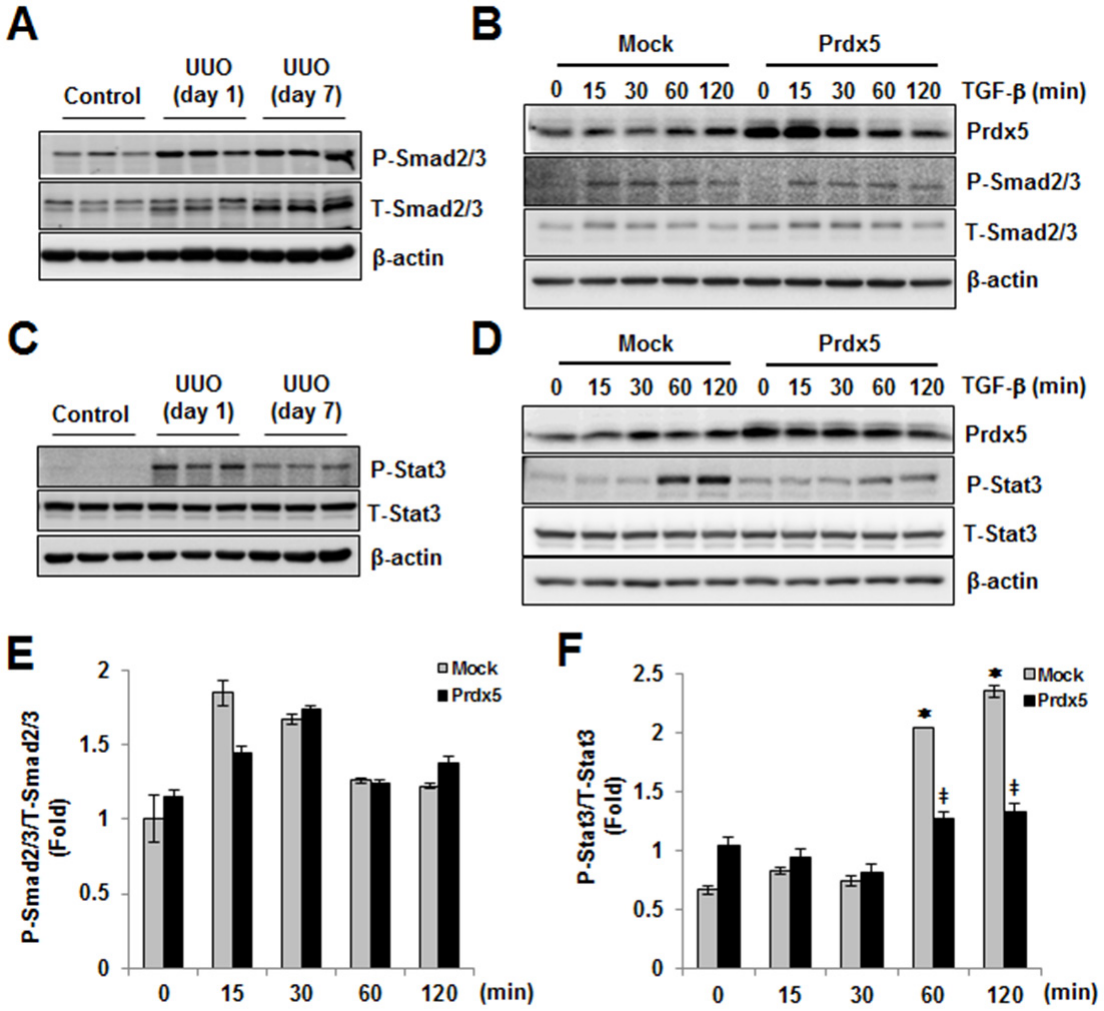
Negative modulation of Prdx5 in TGF-β induced Stat3 activation. To identify signal pathway related to anti-fibrotic effect of Prdx5, Smad2/3 or Stat3 activation were checked in UUO kidney or Prdx5 expressed NRK49F cells for indicated time (0, 15, 30, 60, and 120 min) after TGF-β treatment. Smad2/3 activation in UUO kidney (A) and in mock- or *wild-type Prdx5* transduced NRK49F cells (B) were analyzed by measuring phosphorylation at Ser465/467 in Smad2 and Ser423/425 in Smad3. Total expression of Smad2/3 was evaluated with anti-Smad2/3 antibody. And also, Stat3 activation in UUO kidney (C) and in mock- or *wild-type Prdx5* transduced NRK49F cells (D) were analyzed by measuring phosphorylation at Tyr705 in Stat3. Total expression of Stat3 was evaluated with anti-Stat3 antibody. Bar graphs show mean ratio phospho form to total form of Smad2/3 (E) and Stat3 (F) in mock- or *wild-type Prdx5* transduced NRK49F cells. *p < 0.05 at 0 min vs. the indicated time; ^‡^*p* < 0.05, *Mock* vs. *Prdx5*

TGF-β induced Smad2/3 activation is the canonical pathway in renal fibrosis progression [5]. In fact, phosphorylation of Smad2 at Ser465/467 and Smad3 at Ser423/425 increased in UUO kidneys (Fig 6A). We measured the effect of Prdx5 overexpression on Smad2/3 activation by TGF-β (0, 15, 30, 60, and 120 min). Phosphorylation of Smad2/3 was detected at 15 min in TGF-β treated NRK49F cells, overexpression of Prdx5, however, had no effect (Fig 6B and 6E). Activation of Jak2-Stat3 has also been implicated in renal fibrosis [26]. Like Smad2/3 activation, phosphorylation of Stat3 at Tyr705 increased in the UUO kidney (Fig 6C), but we did not observe activation of the upstream Jak2 signal (data not shown). We then asked whether Prdx5 negatively modulates TGF-β induced activation of Stat3. During TGF-β treatment (0, 15, 30, 60, and 120 min), Stat3 phosphorylation was increased in Mock-transduced NRK49F cells, but not in Prdx5-transduced NRK49F cells (Fig 6D and 6F). These results suggest Prdx5 negatively modulates Stat3 activation to protect against TGF-β induced fibrosis, although Jak2’s involvement was further elucidated.

## Discussion

**Fig 7:**
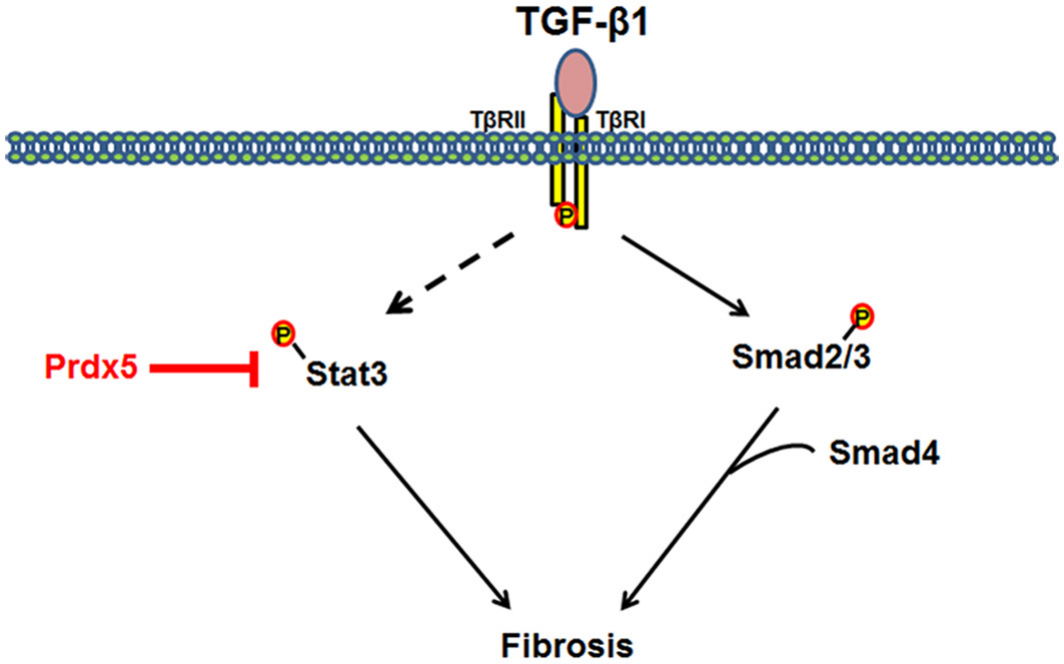
Model mechanism for anti-fibrotic effects of Prdx5. TGF-β mediates the progression of renal fibrosis through several signaling pathways. Overexpression of Prdx5 suppresses activation of Stat3, but has no effect on Smad2/3.

In this study, we firstly demonstrated the involvement of Prdxs isoptypes in renal fibrosis. In Fig 7, we suggested model for physiological function and regulation mechanisms of Prdx5 as anti-fibrotic effector in TGF-β induced renal fibrosis.

Peroxiredoxins are a family of antioxidant enzymes that catalyzes the reduction of hydrogen peroxide. Mammalian cells express six isoforms of Prdxs (Prdx1 to Prdx6), and all six isotypes are expressed in different cell types of kidneys and various subcellular localization [27]. Proximal tubular cells were labelled with Prdx4, whereas distal tubular cells showed abundant labelling with Prdx2, Prdx3, and Prdx5. Papillary tubules and transitional epithelium showed heavy labelling with Prdx5 [27]. At the subcellular level, Prdx1 and Prdx2 were found throughout the cell, while Prdx3 and Prdx5 were primarily mitochondrial, some nuclear labeling was observed. Recent studies point to the importance of Prdxs to exert protective antioxidant effects in various cells [28, 29]. However, the physiological effects and the underlying mechanisms of Prdxs in renal fibrosis have not been fully characterized. In this study, we demonstrated that mitochondria forms of Prdxs (Prdx3 and Prdx5) were decreased associated with renal fibrosis in UUO rat kidneys. Especially, the expression of Prdx5 was rapidly down-regulated at early fibrosis phase. In addition to reduction of Prdx5 in one day UUO kidney, reduction of Prdx3 and Prdx6 were also observed at 7 day of UUO kidney. It suggests that major mitochondrial Prdxs and Prdx6 could be involved in the pathophysiology of renal fibrosis. Prdx3 is a major mitochondrial Prdxs that removes the mitochondrial ROS using thioredoxin-2 as the physiological electron reductant. In fact, mitochondria dysfunction is also involved in aldosterone induced epithelial-mesenchymal transition of renal proximal tubular epithelial cells [30]. Prdx6 is 1-Cys member of Prdxs that possess bifunctional enzyme activity, glutathione peroxidase and phospholipase A 2 (PLA2) activity, by using glutathione instead of thioredoxin as the physiological reductant [31]. Up-regulation of PLA2 activity was related with fibrosing lung diseases, and inhibition of PLA2 activity of Prdx6 reduced NOX2-activated ROS generation in lung ischemia/reperfusion model [32, 33]. However, the involvement of Prdx3 and Prdx6 in renal fibrosis should be further elucidated. Recently it was also reported that the expression of Prdx1 was dramatically reduced in 14 day of UUO kidney and obstructive nephropathic patient kidney, although protein expression of Prdx1 wasn’t changed until 7 day after ureter obstruction in agreement with our data [34]. These findings suggest that Prdx1 may also be related with the pathophysiology of renal fibrosis.

Renal interstitial fibrosis is the hallmark of progressive kidney disease. Tubulointerstitial fibrosis is characterized by the accumulation of extracellular matrix, primarily mediated by fibroblasts in the kidney, making them clinically relevant targets in renal fibrosis. Myofibroblasts represent an activated fibroblast phenotype, which is mainly responsible for the extracellular matrix deposition in tubulointerstitial fibrosis [35]. Profibrotic TGF-β1 is the prime stimulator of this phenotypic activation [36]. We used the rat kidney interstitial fibroblast cells (NRK49F) as an *in vitro* model of α-SMA and fibronectin expression in control and TGF-β1 activated cultures. Expression of α-SMA and fibronectin in NRK49F cells increased after one and three days’ exposure to TGF-β. In association with these changes, Prdx5 expression significantly increased, while other Prdxs isotypes remained unchanged (Prdx1, Prdx2, Prdx3, Prdx6) or mildly decreased (Prdx4) at 5 day of TGF-β treatment in NRK49F cells. These results suggest that Prdx5 may importantly play a role in the pathophysiology of renal fibrosis in kidney interstitial fibroblast cells. In agreement with this notion, there is evidence of the interrelationship between Wnt/fibrosis signaling and Prdx5. Wnt10A over-expression increased not only the level of fibronectin but also Prdx5 expression in a kidney fibroblast cell line [37].

Progressive renal fibrosis was demonstrated in UUO kidneys, which also showed increased levels of premature and mature TGF-β, upregulation of myofibroblast markers fibronectin and α-SMA, and decreased epithelial marker E-cadherin. Based on these findings, we explored the therapeutic potential of Prdx5 as a means of limiting the progression of kidney disease. In this study, overexpression of Prdx5 attenuated the TGF-β induced upregulation of α-SMA and fibronectin in NRK49F cells. Our findings suggest Prdx5 is critically involved in the activation of renal interstitial fibroblasts *in vitro*. Progressive renal interstitial fibrosis is not only the predominant pathological feature of obstructive nephropathy, but is also considered a common final pathway of chronic kidney disease; thus, the Prdx system may play an important role in the pathogenesis of renal interstitial fibrosis in chronic kidney disease. We extended our observations on Prdx5 by using *double mutant Prdx5 (DM Prdx5)*, in which the catalytic *Cys48* and *Cys152* were substituted with *Ser*, and hence mutant Prdx5 does not display peroxidase activity. *Wild type Prdx5* attenuated TGF-β induced upregulation of fibronectin and α-SMA, while *double mutant Prdx5 (DM)* did not affect the TGF-β induced expression of fibronectin and α-SMA compared to *wild type Prdx5*. Accordingly, *WT Prdx5* effectively reduced ROS level elicited by TGF-β, while *DM Prdx5* did not reduce ROS level. These results imply that Prdx5 attenuates TGF-β induced fibrosis in its peroxidase activity dependent manner associated with antioxidant effects.

The involvement of TGF-β in the pathogenesis of fibrosis has been proposed based on its ability to stimulate the production of extracellular matrix proteins and to regulate their metabolism [38]. Among the downstream pathways, Smad2/3 signaling is recognized as a major pathway of TGF-β in kidney fibrosis [39, 40]. In the present study, phosphorylation of Smad2 at Ser465/467 and Smad3 at Ser423/425 was increased in UUO kidneys. Phosphorylation of Smad2/3 was detected at 15 min in TGF-β treated NRK49F cells. Over-expression of Prdx5, however, did not inhibited phosphorylation of Smad2/3 by TGF-β treatment. Among non-canonical TGF-β/Smads pathway, activation of Stat3 has also been implicated in renal fibrosis [26]. Like Smad2/3 activation, phosphorylation of Stat3 at Tyr705 was increased at UUO kidney. We further examined whether over-expression of Prdx5 negatively modulates TGF-β induced activation of Stat3. During TGF-β treatment, activated form of Stat3 was increased in *Mock*-transfected NRK49F cells, but not increased in *Prdx5* transfected NRK49F cells at all. These results suggest that Prdx5 negatively modulates Stat3 activation to protect renal fibrosis. Consequently, we suggested that Prdx5 plays a role as anti-fibrotic effector in the pathogenesis of renal fibrosis, and its regulation mechanism was through by inhibition of Stat3 activation. However, the observed effects of Prdx5 on Stat3 phosphorylation do not necessarily indicate that the Smad2/3 pathway is not affected at all. Further studies are needed to understand the exact interactive mechanisms that couple Prdx5 and unique target genes that contribute to fibrosis development.

The Janus kinase/signal transducers and activators of transcription (Jak/Stat) pathway is a pleiotropic signal cascade for a wide variety of growth factors and cytokines [41]. Enhanced activation of Jak/Stat signaling has been implicated in renal and non-renal cells of various kidney diseases [42]. In fact, high glucose levels induce angiotensin II-dependent activation of Jak2, Stat1, Stat3, and Stat5 and increase of TGF-β and fibronectin synthesis in rat mesangial cells of streptozotocin-induced diabetes [43, 44]. Knockdown of Stat3 (Stat3SA/-) reduces proteinuria, mesangial expansion, glomerular cell proliferation, and macrophage infiltration in comparison to Stat3SA/+ mice in streptozotocin-induced diabetic nephropathy [45]. In the UUO kidney, Stat3 activation in tubulointerstitial cells/myofibroblasts, tubular epithelial cells, and macrophages has also been reported [26, 46]. Indeed, phosphorylation of Stat3 increased in TGF-β treated NRK49F cells, while activation of Jak2 (which functions upstream of Stat) was not observed in our UUO kidney or TGF-β treated NRK49F cells. Thus, Prdx5 negatively regulates Stat3 activation in TGF-β induced fibrosis in NRK49F cells, although upstream molecules involved in Stat3 activation by TGF- β was further elucidated.

Consequently, Prdx5 is an anti-fibrotic effector that sustains renal physiology. Selective inhibition of Stat3 by ectopic expression of Prdx5 could be a therapeutic target in TGF-β induced renal fibrosis.
